# Gender Differences in Case Volume Among Ophthalmology Resident Graduates, 2014-2023

**DOI:** 10.1001/jamaophthalmol.2025.0935

**Published:** 2025-05-01

**Authors:** Susan M. Culican, Misha F. Syed, Yoon Soo Park, Sean O. Hogan

**Affiliations:** 1 Office of Medical Education, Department of Ophthalmology and Visual Neurosciences, University of Minnesota Medical School, Minneapolis; 2 John Sealy School of Medicine, Department of Ophthalmology and Visual Sciences, University of Texas Medical Branch, Galveston; 3 Department of Medical Education, University of Illinois College of Medicine, Chicago; 4 Accreditation Council for Graduate Medical Education, Chicago, Illinois

## Abstract

**Importance:**

A previous multisite study found gender differences in cataract and total surgical volume among US residency programs. Whether that was representative of all ophthalmology residents in the US is unclear.

**Objective:**

To determine whether reported surgical volume among US ophthalmology resident graduates varied by self-reported gender or by underrepresented in medicine (URiM) status.

**Design, Setting, and Participants:**

Retrospective observational longitudinal cohort study of deidentified Accreditation Council for Graduate Medical Education (ACGME) surgical log data for all graduates of US ophthalmology residency programs from the years 2014-2023 compared by self-reported gender or URiM status. Surgical experience was obtained from the ACGME Accreditation Data System resident case log. Data were analyzed from November 2023 to April 2024.

**Main Outcomes and Measures:**

The main outcome was a difference in mean reported surgical volume between comparison groups by gender or URiM status for cataract or total surgical procedures. The secondary outcomes were differences for other surgical categories by gender or URiM status.

**Results:**

Of 4811 resident graduates, 41.6% (1999) were female and 58.4% were male (2812); 7.1% (343) self-identified as URiM. Cataract cases had a mean difference of −4.4% (−8.3 of 189.2) (95% CI, −6.4% to −2.4%; *P* < .001) fewer surgeries for female residents than male residents over the 10-year study, and total procedures had a mean difference of −7.4% (−43.4 of 587.3) (95% CI, −9.7% to −5.1%; *P* < .001) fewer surgeries for female than male residents over the 10-year study. URiM status over the same time period was not associated with a difference in cataract surgeries but was associated with a mean difference of −5.3% (−31.5 of 587.3) (95% CI, −9.8% to −0.9%; *P* = .02) fewer total procedures.

**Conclusions and Relevance:**

In this study, female residents reported fewer cataract procedures than male residents from 2014-2023. Female and URiM residents reported fewer total procedures than their nonfemale or non-URiM colleagues. Future studies to assess the causes of these disparities are warranted.

## Key Points

**Question:**

Do women or underrepresented in medicine (URiM) trainees report disproportionately fewer cataract or total operative procedures than males or non-URiM trainees during residency training?

**Findings:**

In this cohort study including 4811 ophthalmology resident graduates from 2014 through November 2023, women reported fewer cataract and total surgical cases than men. URiM trainees did not demonstrate a reduction of cataract experience during training but performed fewer total procedures than non-URiM residents.

**Meaning:**

The results of this study suggest a gender and URiM disparity in surgical experience during ophthalmology residency training in the US over the last decade.

## Teaser

This cohort study assesses whether ophthalmology resident trainees who are underrepresented in medicine (URiM) reported equivalent surgical experiences to non-URiM trainees in the same cohort.

## Introduction

A prior multisite study^1^ that included approximately 20% of US residency programs reported disparity in ophthalmology residents’ surgical volume based on surgeon gender for cataract surgery and total surgical procedures where women logged fewer cases than men. Our goal with the present study was to determine whether the finding in this sample could be explained by selection bias or whether the finding was representative across all resident trainees in Accreditation Council for Graduate Medical Education (ACGME)–accredited programs. In addition, we explored whether trainees who are underrepresented in medicine (URiM) reported equivalent surgical experiences to non-URiM trainees in the same cohort.

## Methods

### Study type

This is a national retrospective longitudinal cohort study of surgical experience for graduates of ACGME-accredited ophthalmology residency programs from the years 2014-2023. This study followed the Strengthening the Reporting of Observational Studies in Epidemiology (STROBE) reporting guideline. The main outcome was a difference in mean reported surgical volume between comparison groups by gender or URiM status for cataract or total surgical procedures. The secondary outcomes were differences for other surgical categories by gender or URiM status.

### Setting and Participants

This cohort includes all graduates of ACGME-accredited ophthalmology residency training programs for the years 2014-2023. Surgical experience was obtained from the ACGME Accreditation Data System resident case log. The dataset does not contain information about age. The race and ethnicity data were the most recent race and ethnicity data that the Association of American Medical Colleges (AAMC) has on the trainees in this study. For the vast majority of trainees, the most recent data will be the self-reported data from the AAMC Electronic Residency Application Service, with additional race and ethnicity reporting coming from other AAMC applications (such as from the American Medical College Application Service). All data were self-reported by residents to the AAMC or the ACGME. Analysis of the deidentified dataset was deemed not human studies research by the institutional review boards at the University of Minnesota and the University of Illinois Chicago and exempt from institutional review board approval at the University of Texas Medical Branch.

### Statistical Analysis

Deidentified data for each required procedural category (eTable 1 in Supplement 1) over the 10-year study period were analyzed from November 2023 to April 2024. URiM was defined as those racial and ethnic populations that are underrepresented in the medical profession relative to their numbers in the general population based on 2023 categorizations from the AAMC.^2^ For the purpose of this study, those populations included trainees who self-identified as one or more of the following race and ethnicity categories (alone or in combination with any other race and ethnicity category): American Indian or Alaska Native; Black or African American; Hispanic, Latino, or of Spanish Origin; or Native Hawaiian or Other Pacific Islander. Non-URiM designations included White or Caucasian and Asian.

Descriptive statistics were used to measure trends over the 10-year period. Bivariate comparisons were conducted using *t* tests for gender differences and URiM differences by category and by graduating cohort. All *P* values were 2-sided and not adjusted for multiple analyses. Statistical significance was set at *P* < .05. To examine the aggregate effect estimates of the 10-year longitudinal period, conditional growth curve analyses were conducted using mixed-effects regression, clustering by year and program. We specified main effect estimates and interaction effect estimates between gender and URiM status to examine baseline group differences and differences over time. Data compilation and analyses were conducted using Stata 18/MP (StataCorp LLC).

## Results

Of 4811 resident graduates, 41.6% (1999) were female and 58.4% were male (2812); 7.1% (343) self-identified as URiM. The total number of graduates by gender and URiM status in each year of the 10-year study period is listed in Table 1. The total number of graduates increased by 10.3% from 458 in 2013-2014 to 505 in 2022-2023. The proportion of women in each year ranged from 37.6% (188 of 500) in 2020-2021 to 44.3% (224 of 506) in 2021-2022 (average, 41.6%) and was stable. The number of URiM learners increased 4-fold during the study period from 13 (2.8% of 458) in academic year 2014 to 57 (11.3% of 505) in academic year 2023 (Table 1). The proportion of female URiM trainees fluctuated over the 10 years of the study from 15.4% to 70.4%, with a mean (SD) of 53.9% (49.9%) (185 of 343).

**Table 1.** Distribution of Resident Trainees by Self-Identified Gender and URiM Status Over the 10-Year Study Period, 2014-2023

Completion year	Total No.	Trainees, No. (%)		
		Male (%)	Female (%)	URiM (%)
2013-2014	458	262 (57.2)	196 (42.8)	13 (2.8)
2014-2015	461	261 (56.6)	200 (43.4)	22 (4.8)
2015-2016	468	261 (55.8)	207 (44.2)	20 (4.3)
2016-2017	472	277 (58.7)	195 (41.3)	27 (5.7)
2017-2018	484	289 (59.7)	195 (40.3)	28 (5.8)
2018-2019	466	282 (60.5)	184 (39.5)	43 (9.2)
2019-2020	491	286 (58.2)	205 (41.8)	37 (7.5)
2020-2021	500	312 (62.4)	188 (37.6)	45 (9.0)
2021-2022	506	282 (55.7)	224 (44.3)	51 (10.1)
2022-2023	505	300 (59.4)	205 (40.6)	57 (11.3)
Total 2014-2023	4811	2812 (58.4)	1999 (41.6)	343 (7.1)

Abbreviation: URiM, underrepresented in medicine.

Over the study period, the changes in the total volume of logged surgeries varied by category. Eighteen categories of procedures were tracked in the surgical log. Of these, 8 categories, including cataract and total procedures, increased over the study period. Six categories were stable over 10 years and 4 categories saw declines (eTable 2 in Supplement 1). All group means (female, male, URiM, non-URiM) exceeded the ACGME-required surgical minima for all procedural categories (Table 2).

**Table 2.** ACGME Surgical Categories and Reported Surgical Experience by Trainee Type, 2014-2023

Procedural category	ACGME-required surgical minimum for graduating residents^a^	Resident group, No., mean (SD)			
		Female	Male	URiM	Non-URiM
Cataract	86	184.4 (63.2)	192.7 (67.9)	184.8 (63.0)	189.6 (66.3)
Glaucoma–filtering/shunting procedures	5	11.8 (8.7)	12.1 (8.8)	12.2 (8.9)	12.0 (8.8)
Globe trauma	4	9.3 (5.5)	9.7 (5.7)	9.2 (5.2)	9.6 (5.6)
Intravitreal injection	10	120.8 (128.5)	133.6 (138.5)	120.7 (137.9)	128.9 (134.3)
Keratoplasty	5	10.0 (6.3)	10.1 (6.6)	10.1 (6.6)	10.1 (6.4)
Keratorefractive surgery	6	12.8 (13.7)	16.5 (24.3)	12.6 (14.5)	15.1 (21.1)
Laser surgery–laser iridotomy	4	14.3 (11.0)	14.4 (10.5)	13.4 (9.6)	14.4 (10.8)
Laser surgery–laser trabeculoplasty	5	15.1 (14.1)	15.7 (14.0)	15.3 (14.6)	15.4 (14.0)
Laser surgery–PRP	10	37.9 (51.0)	44.5 (55.8)	33.4 (41.0)	42.4 (54.7)
Laser surgery–YAG capsulotomy	5	19.6 (12.9)	23.4 (16.2)	21.0 (14.3)	21.8 (15.1)
Oculoplastic and orbit	28	66.4 (35.4)	70.1 (39.3)	63.6 (33.9)	69.0 (38.0)
Oculoplastic and orbit–chalazion excision	3	8.1 (5.7)	8.3 (5.9)	8.0 (7.1)	8.2 (5.7)
Oculoplastic and orbit–eyelid laceration	3	10.2 (7.3)	10.5 (8.2)	9.8 (7.3)	10.4 (7.9)
Oculoplastic and orbit–ptosis/blepharoplasty	3	9.5 (12.8)	9.8 (12.8)	10.0 (13.3)	9.7 (12.8)
Pterygium/conjunctival and other cornea	3	9.7 (7.2)	9.9 (7.8)	10.7 (8.4)	9.8 (7.5)
Retinal vitreous	10	25.7 (17.4)	28.6 (19.9)	29.4 (20.4)	27.2 (18.8)
Strabismus	10	24.2 (14.9)	24.0 (14.6)	21.6 (13.6)	24.3 (14.8)
Total	NA	561.9 (223.1)	605.4 (246.5)	558.1 (235.7)	589.6 (238.0)
Total other (total minus cataract)^b^	NA	405.4 (198.7)	441.3 (219.5)	401.0 (211.4)	428.3 (211.7)

Abbreviations: ACGME, Accreditation Council for Graduate Medical Education; NA, not applicable; PRP, panretinal photocoagulation; URiM, underrepresented in medicine; YAG, yttrium aluminum garnet.

^a^ Surgical minimum requirements are established by the ACGME Ophthalmology Review Committee to ensure programs provide sufficient surgical experience for resident graduates.

^b^ Because cataract surgery represents approximately one-third of the total volume of surgical procedures, an additional category of total other (total minus cataract) procedures is included.

The distribution of reported surgical case volume by gender demonstrated a disparity between female and male residents in numerous surgical categories (Figure, A). Being female was associated with fewer primary surgeon cataract procedures (10-year mean [SD]: female, 184.4 [63.2]; male, 192.7 [67.9]) and fewer total procedures (10-year mean [SD]: female, 561.9 [223.1]; male, 605.4 [246.5]). For cataract surgery, female residents had a mean difference of −4.4% (−8.3 of 189.2) fewer cases (95% CI, −6.4% to −2.4%; *P* < .001) than male residents, and this disparity persisted over time (Figure, A; eTable 3 in Supplement 1). The total number of cases had a mean difference of −7.4% (−43.4 of 587.3) fewer procedures for women than for men (95% CI, −9.7% to −5.1%; *P* < .001). Secondary outcome analyses demonstrated gender disparity across additional surgical procedures, including globe trauma, intravitreal injection, keratorefractive surgery, panretinal photocoagulation laser, YAG (yttrium aluminum garnet) capsulotomy, oculoplastic and orbit, and retina and vitreous (Figure, A; Table 3). The smallest mean disparity across all categories was for cataract surgery and the largest was for keratorefractive surgery (mean, −25.2%; 95% CI, −33.1% to −17.3%).

**Figure.**
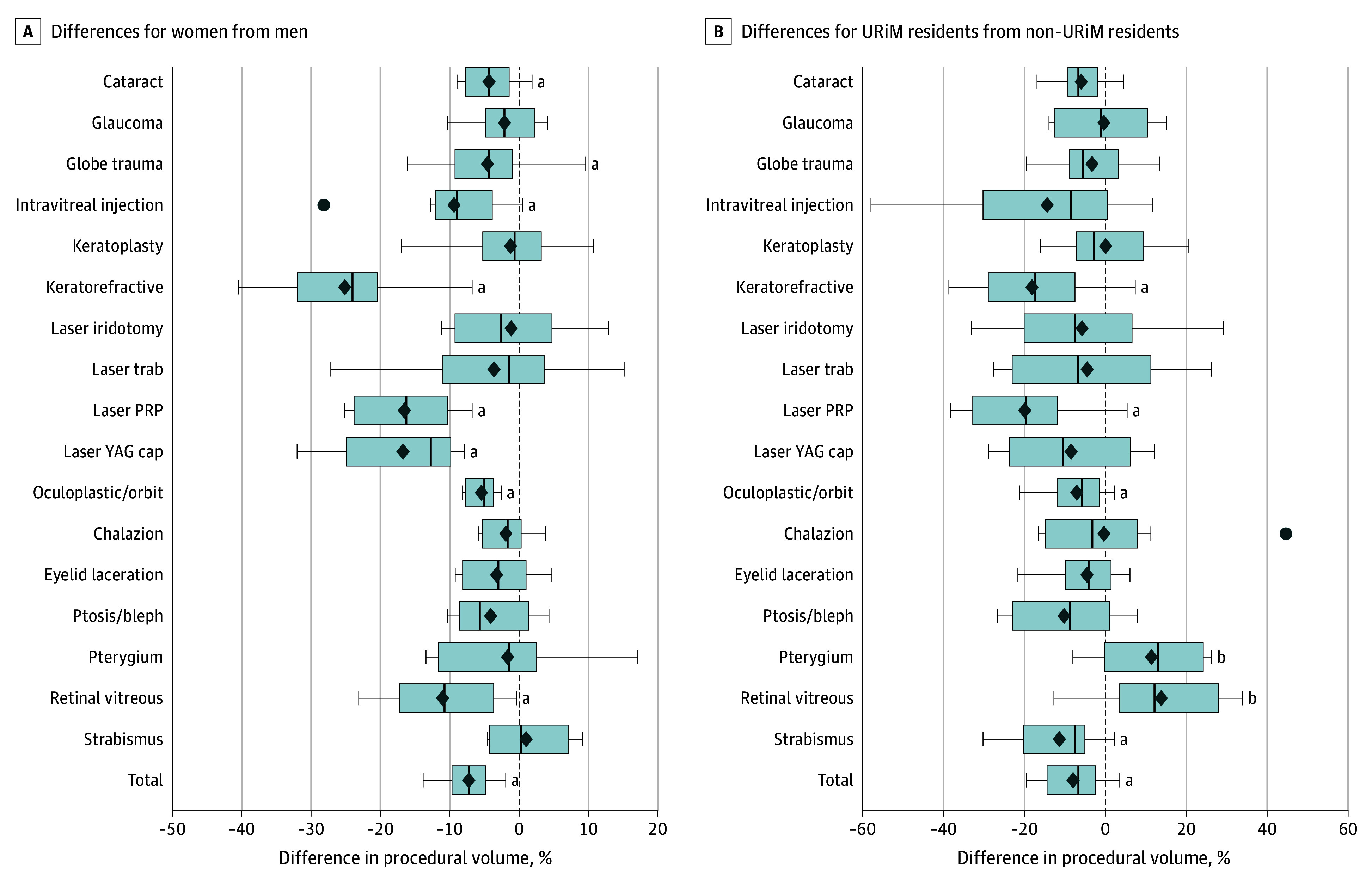
Differences in Procedural Volume Between Comparison Groups Over 10 Years by Ophthalmology Surgical Case Log Category, 2014-2023 Differences are normalized as a percentage of the mean value for all learners in a given year. Box and whisker plots depict 10 years of comparison by procedure for women:men (A) and URiM:non-URiM (B). Boxes indicate the 25th percentile, median, and 75th percentile. The diamonds denote the mean of differences over 10 years. The filled blue circles represent outliers. Bleph indicates blepharoplasty; PRP, panretinal photocoagulation; trab, trabeculoplasty; URiM, underrepresented in medicine; and YAG cap, yttrium aluminum garnet capsulotomy. ^a^Lower procedural volume for women or URiM trainees (*P* < .05). ^b^Higher procedural volume for URiM than for non-URiM trainees.

**Table 3.** Differences Between Reported Surgical Case Volume By Gender^a^

Category	Mean difference	Mean difference, % (95% CI)^b^	*P* value
Cataract	−8.3	−4.4 ( −6.4 to −2.4)	<.001
Glaucoma–filtering/shunting procedures	−0.3	−2.5 ( −6.7 to 1.7)	.24
Globe trauma	−0.4	−4.6 ( −7.9 to −1.2)	.007
Intravitreal injection	−12.9	−10.0 (−16.0 to −4.0)	.001
Keratoplasty	−0.1	−1.2 (−4.9 to 2.4)	.51
Keratorefractive surgery	−3.8	−25.2 (−33.1 to −17.3)	<.001
Laser surgery–laser iridotomy	−0.1	−0.8 ( −5.1 to 3.4)	.70
Laser surgery–laser trabeculoplasty	−0.6	−4.1 (−9.3 to 1.1)	.12
Laser surgery–panretinal laser photocoagulation	−6.6	−15.8 (−23.2 to −8.4)	<.001
Laser surgery–YAG capsulotomy	−3.8	−17.4 (−21.3 to −13.5)	<.001
Oculoplastic and orbit	−3.7	−5.3 (−8.5 to −2.2)	.001
Oculoplastic and orbit–chalazion excision	−0.2	−2.1 (−6.1 to 2.0)	.33
Oculoplastic and orbit–eyelid laceration	−0.4	−3.5 (−7.8 to 0.8)	.11
Oculoplastic and orbit–ptosis/blepharoplasty	−0.3	−3.1 (−10.7 to 4.4)	.42
Pterygium/conjunctival and other cornea	−0.1	−1.5 (−5.9 to 2.9)	.52
Retinal vitreous	−2.9	−10.7 (−14.6 to −6.7)	<.001
Strabismus	0.3	1.1 (−2.4 to 4.6)	.54
Total	−43.4	−7.4 (−9.7 to −5.1)	<.001
Total other (total minus cataract)^c^	−36.0	−8.4 (−11.3 to −5.6)	<.001

Abbreviation: YAG, yttrium aluminum garnet.

^a^ Negative values indicate fewer procedures reported by female than male residents.

^b^ Percentage difference (mean difference/mean total) between female and male groups for each surgical category over the 10 year study period, 2014-2023.

^c^ Because cataract surgery represents approximately one-third of the total volume of surgical procedures, an additional category of total other (total minus cataract) procedures is included.

Case volume distribution by URiM status varied (Figure, B; eTable 4 in Supplement 1). There were no differences in mean reported cataract surgical volume for URiM trainees, but URiM status was associated with fewer total procedures than for non-URiM residents (mean [SD]: URiM, 558.1 [235.7]; non-URiM, 589.6 [238.0]). URiM residents reported a mean difference of −5.3% (−31.5 of 587.3) (95% CI, −9.8% to −0.9%; *P* = .02) fewer total procedures than their non-URiM colleagues. Secondary outcomes analysis demonstrated reduced numbers of keratorefractive, YAG capsulotomy, oculoplastic and orbit procedures, and strabismus cases for URiM trainees (Table 4). Two categories demonstrated higher procedural volume for URiM trainees: pterygium/conjunctival procedures (mean percentage difference, 10.0%; 95% CI, 1.6%-18.4%; *P* = .02) and retina/vitreous procedures (mean percentage difference, 7.8%; 95% CI, 0.2%-15.4%; *P* = .04).

**Table 4.** Differences Between Reported Surgical Case Volume by URiM Status^a^

Category	Mean difference	Mean difference, % (95% CI)^b^	*P* value
Cataract	−4.7	−2.5 ( −6.3 to 1.3)	.20
Glaucoma–filtering/shunting procedures	0.2	1.4 (−6.6 to 9.4)	.73
Globe trauma	−0.3	−3.6 (−10.0 to 2.9)	.28
Intravitreal injection	−8.1	−6.4 (−17.9 to 5.2)	.28
Keratoplasty	0.0	0.1 (−6.9 to 7.2)	.97
Keratorefractive surgery	−2.6	−17.2 (−32.4 to −2.0)	.03
Laser surgery–laser iridotomy	−1.0	−7.0 (−15.2 to 1.2)	.09
Laser surgery–laser trabeculoplasty	−0.1	−0.8 (−10.8 to 9.2)	.87
Laser surgery–panretinal photocoagulation	−9.0	−21.6 (−35.8 to −7.4)	.003
Laser surgery–YAG capsulotomy	−0.9	−4.1 (−11.7 to 3.5)	.29
Oculoplastic and orbit	−5.3	−7.8 (−13.8 to −1.7)	.01
Oculoplastic and orbit–chalazion excision	−0.2	−2.6 (−10.4 to 5.3)	.52
Oculoplastic and orbit–eyelid laceration	−0.6	−6.1 (−14.4 to 2.2)	.15
Oculoplastic and orbit–ptosis/blepharoplasty	0.3	3.4 (−11.1 to 17.9)	.65
Pterygium/conjunctival and other cornea	1.0	10.0 (1.6 to 18.4)	.02
Retinal vitreous	2.1	7.8 (0.2 to 15.4)	.04
Strabismus	−2.6	−10.9 (−17.7 to −4.2)	.002
Total	−31.5	−5.4 (−9.8 to −0.9)	.02
Total other (total minus cataract)^c^	−27.3	−6.4 (−11.9 to −0.9)	.02

Abbreviations: URiM, underrepresented in medicine; YAG, yttrium aluminum garnet.

^a^ Negative values indicate fewer procedures reported by URiM than non-URiM residents.

^b^ Percentage difference (mean difference/mean total) between URiM and non-URiM groups for each surgical category over the 10-year study period, 2014-2023.

^c^ Because cataract surgery represents approximately one-third of the total volume of surgical procedures, an additional category of total other (total minus cataract) procedures is included.

## Discussion

A prior study^1^ described disparity in ophthalmology residents’ surgical volume based on surgeon gender for cataract surgery and total surgical procedures where women logged fewer cases than men. In this cohort, consisting of a limited number of programs in the US, the surgical gap increased over time, and the difference could not be explained by parental leaves, which are more commonly taken by women thus potentially reducing clinical contact time.^1^ Our goal with this study was to determine whether this cohort was representative across all resident trainees in ACGME-accredited programs. Indeed, we found that the mean reported cataract and total surgical volume was lower for women than for men and sustained over the 10 years of our study. Women reported fewer surgical procedures in 7 additional procedural categories. A parallel analysis of URiM trainees did not demonstrate differences in reported cataract experience but did illustrate disparity in total procedural volume and in 4 other surgical areas. Despite surgical volume disparities between groups, the mean surgical volume for all comparison groups exceeded the ACGME-required surgical minima.

The reasons for the disparities in ophthalmology resident surgery during residency training should be explored. One hypothesis already mentioned is that parental leave could disproportionately affect women and impact surgical volume. However, Gong et al^1^ found that parental leave taken by women did not affect their surgical volume. Other studies of ophthalmology training programs in India and Australia and New Zealand similarly did not demonstrate a correlation with parental leave and lower surgical volume by gender.^3,4^ Gender disparity in surgical volume not explained by parental leave has also been described in gastroenterology, general surgery, and otolaryngology training programs.^5,6,7^

When looking at subspecialty case volume disparity, a possible explanation is that residents who are pursuing subspecialty training are more motivated to complete extra procedures in their area of interest. Because some subspecialties are historically more heavily male represented than in ophthalmology as a whole, the disparity may reflect a selection bias,^8,9^ so it follows that the residents with high case volumes in these specialties are more likely male. Self-reported demographic data to the American Board of Ophthalmology for the 2023 diplomates demonstrate a persistent deficit in the number of practicing women ophthalmologists (26%) compared with the number of women in training (41%) (American Board of Ophthalmology, unpublished 2023 data, July 31, 2024). However, in some subspecialties representation better reflects the training distribution (36% comprehensive ophthalmology, 38% oculoplastics, 35% cornea). While the retina subspecialty is heavily male dominated (19% female) and keratorefractive subspecialty is heavily male dominated (14% female), pediatric ophthalmology (55%) and the traditionally nonsurgical subspecialties of neuro-ophthalmology (56%), uveitis (51%), and medical retina (47%) have a disproportionately high representation of women (eTable 5 in Supplement 1). A 2021 study exploring the surgical volume among vitreoretinal fellows demonstrated that male vitreoretinal fellows reported more procedures than their female colleagues and women vitreoretinal fellows reported completing fewer endolaser, internal limiting membrane peel, and cryoretinopexy procedures in the second year of fellowship training.^10^ This suggests that even among specialty-motivated trainees, there remains a gender disparity in case volume.

The disparities identified for URiM trainees are more difficult to interpret. There were half as many categories where URiM trainees reported fewer cases than female trainees (5 for URiM trainees and 9 for female trainees). Surprisingly, 2 areas demonstrated increased case numbers for URiM trainees. Because of small sample sizes, we were unable to explore gender by URiM interaction. However, we did find that the proportion of female trainees in the URiM group was higher than in the total cohort and that the proportion of URiM trainees increased over time, while the proportion of women remained stable. Given the small number of URiM learners and unequal sample sizes, this gender imbalance could contribute to the differences observed.

The finding that URiM trainees report fewer surgical total procedures is consistent with other reports of disparities in surgical training based on URiM status. In general surgery, several studies have demonstrated consistently lower surgical case volume among URiM residents vs non-URiM residents. Specifically, Eruchalu et al^11^ found at a single institution that both female and URiM residents performed fewer assistant and primary surgical cases than male non-URiM residents. A follow-up study demonstrated persistently lower volume at graduation for Black residents than nonminority residents in a multi-institutional analysis.^12^ Other surgical specialties have shown similar differences. In obstetrics/gynecology, a single-institution study demonstrated an average fewer number of total procedures as well as fewer number of abortion procedures completed among URiM residents compared with non-URiM trainees.^13^

Other factors that might account for the lower female case volume observed in our study include trainee confidence (both self-confidence and others’ confidence in trainees) and faculty granting of surgical autonomy during training.^14,15,16,17,18^ Meyerson et al^19^ demonstrated that resident gender is an independent predictor of operative autonomy among thoracic surgery residents. Hoops et al^17^ reported similar independent gender-based differences in resident surgical autonomy during laparoscopic procedures, but there was no measured difference in surgical skill between genders. It follows that if faculty grant less autonomy to female residents over the course of training that they will have fewer surgical opportunities.^19^ Fonseca et al^16^ measured resident operative confidence at the conclusion of general surgery training and showed that women residents are less confident than their male counterparts overall and that both male sex and increased surgical volume were independently associated with a higher level of reported self-confidence. Stanek et al^18^ demonstrated in a simulation study with plastic surgery trainees that women reported lower self-confidence despite the study showing no gender-related differences in objective performance. Whether lower confidence is a cause of, or results from, the lack of granted autonomy is unclear. However, the perception by both trainees (women report less confidence) and faculty (faculty grant less autonomy) that women are less surgically able contributes to a damaging feedback loop that results in lower surgical volume and subsequent undertraining of female surgeons.

An added contributing factor may be societal gender norms and associated implicit gender bias.^20^ When men exhibit confidence and assertiveness, the behavior is gender concordant and perceived positively. Women with the same behavior, which is gender nonconcordant, may be labeled as arrogant, self-promoting, or conceited.^21^ Fear of this repercussion may result in women being more passive and not advocating for themselves.^19^ Indeed, women report fearing they are less likable if they behave assertively. Hesitancy can be perceived as lower confidence, which then affects one’s perceived competence.^15^

There is evidence that the aforementioned gaps in training persist into clinical practice. A study of practicing cataract surgeons found that female ophthalmologists perform fewer than half the number of cataract surgeries than their male counterparts, even accounting for number of years in practice and regardless of whether subspecialty training was completed.^22^ There was an even greater gender volume disparity when French et al^22^ analyzed the number of surgeries done among high-volume surgeons (defined as >1000 cataract surgeries per year): only 6 women between 2005-2012 reached this threshold. In the Feng et al^23^ study, cataract surgical data from Medicare beneficiaries in 2017 were analyzed by surgeon gender for predictors of cataract volume. The authors found that female ophthalmologists performed fewer cataract surgeries than their male colleagues, even after accounting for differences in clinical productivity and physician experience.^23^ This disparity persisted across all geographic regions in the US. A systematic review by Rousta et al^20^ discussed a Canadian study showing further volume differences among women and men ophthalmologists in terms of the number of operating days per month, which demonstrated that 49% of the women surgeons had 2 or more operating days per month vs 64% of the men.

Despite the documented difference in surgical volume in training and in practice, there is no evidence that clinical outcomes are adversely affected. In fact, women are shown to have equivalent skill levels and patient outcomes and to have reduced rates of being sued^20^ in clinical practice. Gupta et al^4^ and Gill et al^3^ demonstrated comparable surgical outcomes and complication rates for female residents regardless of surgical volume.

If surgical outcomes are unaffected by lower volume, why is it important to address gender disparity? It is well documented that there is a pay gap for women in ophthalmology.^20,24,25,26^ This pay differential has historically been justified by procedural volume (higher volume, higher pay). If female trainees are disadvantaged with lower-volume training from residency onward, we handicap women from the start, making it difficult to overcome the inequity.^20^ In addition, an ophthalmology workforce shortage combined with an aging population requires increased clinical capacity by practicing ophthalmologists. With 40% of the current graduates being female, it is imperative to maximize clinical throughput to address this burgeoning need.

### Limitations

This study has limitations. Our dataset was limited to ACGME case volume and could not address potential causes that might explain the disparities identified. Because our dataset is a self-reported log of procedural experience, it is possible that women disproportionately underreport, or that men overreport, their surgical experience. However, there is no evidence to suggest that inaccurate reporting varies by gender. Further, our data analysis did not include program-level data to explore the possibility that different programs have different surgical opportunities and that interprogram differences could account for the disparity. For example, a disproportionate representation of men in higher surgical-volume programs and/or women in programs with lower case volumes would skew the population data toward lower overall numbers for women vs men. With regard to the URiM differences, we were limited by small and unequal sample sizes and unable to explore potential gender by URiM interactions. We note that while gender distribution was stable over the 10-year study period, the proportion of URiM trainees increased over time and was disproportionately female. Thus, URiM differences in case volume over time may be confounded due to concurrent changes in demographics and the small number of URiM trainees at early time points. Furthermore, because URiM trainee experience reflects only a small number of programs, it is unknown whether the programs that train URiM learners are more or less reflective of experiences across all programs.

Factors that account for the disparities may inform solutions to address them. For example, if the reason that the URiM group had fewer procedures is explained by a gender difference, then solutions might be focused on gender disparities more generally. Were a program-level analysis to show that gender disparities could be explained by disproportionate gender representation in high-volume and low-volume programs, understanding the factors that contribute to the selection of programs by learners and the recruitment of gender-diverse classes by programs might be explored. If program-level analysis found that disparities persist across rather than between programs, exploring local factors, such as potential implicit bias, would be indicated.

## Conclusions

This analysis of all graduates of ACGME-accredited ophthalmology residency programs over 10 years has demonstrated that despite exceeding all ACGME-required minima, women reported fewer cataract procedures and female and URiM residents reported fewer total surgical cases than their male or non-URiM counterparts. To our knowledge, there is no evidence to suggest that this disparity results in inadequate preparation for female and URiM ophthalmologists entering practice. Because surgical volume dictates compensation, undertraining residents may have career-long implications for pay equity. In addition, patient access to care could benefit if women in the ophthalmology workforce achieve comparable output to their male colleagues to better serve the patient population. These results warrant additional studies exploring the contribution of program-level differences and the intersectional impact of race and gender to test hypotheses about the potential causes and contributing factors to these disparities.

## Notes

Supplement 1. **eTable 1.** Surgeon Role Minimum Requirements **eTable 2.** Changes in Case Volume Over the 10 Year Study Period **eTable 3.** Annual Longitudinal Change in Procedural Disparities for Female Residents **eTable 4.** Annual Longitudinal Change in Procedural Disparities for URiM Residents **eTable 5.** Distribution of Active American Board of Ophthalmology Diplomates for Those Who Self-Reported Gender With Certification and Practice Emphasis

Supplement 2.
Data Sharing Statement
