# Supplementary material for: Gender Differences in Case Volume Among Ophthalmology Resident Graduates, 2014-2023
Source: JAMA Ophthalmol. 2025 May 1;143(6):490–7. doi: 10.1001/jamaophthalmol.2025.0935 (PMC12046517; doi:10.1001/jamaophthalmol.2025.0935)
Supplement: Supplement 1. — eTable 1. Surgeon Role Minimum Requirements eTable 2. Changes in Case Volume Over the 10 Year Study Period eTable 3. Annual Longitudinal Change in Procedural Disparities for Female Residents eTable 4. Annual Longitudinal Change in Procedural Disparities for URiM Residents eTable 5. Distribution of Active American Board of Ophthalmology Diplomates for Those Who Self-Reported Gender With Certification and Practice Emphasis [file jamaophthalmol-e250935-s001.pdf]

Supplemental Online Content

Culican SM, Syed MF, Park YS, Hogan SO. Gender differences in case volume among ophthalmology resident graduates 2014-2023. *JAMA Ophthalmol.* Published online May 1, 2025. doi:10.1001/jamaophthalmol.2025.0935

eTable 1: Surgeon Role Minimum Requirements
eTable 2: Changes in Case Volume Over the 10 Year Study Periodw
eTable 3: Annual Longitudinal Change in Procedural Disparities for Female Residents
eTable 4: Annual Longitudinal Change in Procedural Disparities for URiM Residents
eTable 5: Distribution of Active American Board of Ophthalmology Diplomates for Those Who Self-Reported Gender With Certification and Practice Emphasis

© 2025 Culican SM et al. *JAMA Ophthalmology*.

eTable 1: Surgeon Role minimum requirements. (S) surgeon only. (S+A) surgeon plus assistant.

Category from ACGME Case log Feb 2022	Role
Cataract	S
Glaucoma - Filtering/shunting procedures	S
Globe Trauma	S
Intravitreal Injection	S
Keratoplasty	S+A
Keratorefractive Surgery	S+A
Laser Surgery - Laser iridotomy	S
Laser Surgery - Laser trabeculoplasty	S
Laser Surgery - Panretinal laser photcoagulation	S
Laser Surgery - YAG Capsulotomy	S
Oculoplastic and Orbit	S
Oculoplastic and Orbit - Chalazion Excision	S
Oculoplastic and Orbit - Eyelid laceration	S
Oculoplastic and Orbit - Ptosis/blepharoplasty	S
Pterygium/conjunctival and other cornea	S
Retinal Vitreous	S+A
Strabismus	S
Total	S+A

© 2025 Culican SM et al. *JAMA Ophthalmology*.

eTable 2: Changes in case volume over the 10 year study period. Time (slope) coefficients (Coef) show mean annual increases (positive slope) and mean annual decreases (negative slope) in different procedure volumes over time. Intercept coefficient (Coef) estimates the baseline number of cases logged for each category in 2014.

Category	Time (Slope)			Intercept		
	Coef	SE	*P*-value	Coef	SE	*P*-value
Cataract	3.13	1.51	.04	178.64	8.09	< .001
Glaucoma - Filtering/shunting procedures	0.19	0.08	.02	11.26	0.45	< .001
Globe Trauma	0.19	0.05	< .001	8.85	0.26	< .001
Intravitreal Injection	7.95	0.96	< .001	97.66	5.17	< .001
Keratoplasty	-0.21	0.05	< .001	11.09	0.24	< .001
Keratorefractive Surgery	-0.01	0.18	.96	16.68	0.94	< .001
Laser Surgery - Laser iridotomy	-0.42	0.12	< .001	16.37	0.64	< .001
Laser Surgery - Laser trabeculoplasty	0.30	0.11	.01	14.31	0.61	< .001
Laser Surgery - Panretinal laser photcoagulation	-1.23	0.40	.002	50.59	2.15	< .001
Laser Surgery - YAG Capsulotomy	0.72	0.11	< .001	20.07	0.61	< .001
Oculoplastic and Orbit	-0.36	0.34	.29	72.05	1.84	< .001
Oculoplastic and Orbit - Chalazion Excision	-0.03	0.05	.50	8.42	0.26	< .001
Oculoplastic and Orbit - Eyelid laceration	0.05	0.06	.33	10.32	0.30	< .001
Oculoplastic and Orbit - Ptosis/blepharoplasty	1.34	0.43	.002	3.74	2.29	.10
Pterygium/conjunctival and other cornea	0.04	0.06	.47	9.64	0.32	< .001
Retinal Vitreous	-0.39	0.13	.002	30.24	0.68	< .001
Strabismus	-0.06	0.12	.61	24.39	0.64	< .001
Total	9.85	2.72	< .001	561.88	14.61	< .001
Total Other (Total minus cataract)	0.72	0.11	< .001	20.07	0.61	< .001

© 2025 Culican SM et al. *JAMA Ophthalmology*.

eTable 3: Annual longitudinal change in procedural disparities for female residents. At baseline women reported fewer procedures for cataract, keratorefractive, laser surgery PRP and YAG capsulotomy, oculoplastic and orbit, retina vitreous, total surgeries and total non-cataract surgery (female baseline differences). These differences persisted over the course of the study period. Laser iridotomy initially was not associated with a gender difference in 2014 but became increasingly disparate annually over the 10 year study period (female x time).

Category	Female (Baseline Difference)			Female x Time (Difference over Time)		
	Coef	SE	*P*-value	Coef	SE	*P*-value
Cataract	-10.75	3.51	.002	0.70	0.65	.28
Glaucoma - Filtering/shunting procedures	-0.21	0.48	.66	-0.01	0.09	.89
Globe Trauma	-0.30	0.31	.33	-0.02	0.06	.68
Intravitreal Injection	-11.33	7.28	.12	-0.04	1.35	.98
Keratoplasty	-0.34	0.35	.34	0.04	0.07	.54
Keratorefractive Surgery	-2.70	1.13	.02	-0.22	0.21	.29
Laser Surgery - Laser iridotomy	0.99	0.58	.09	-0.24	0.11	.02
Laser Surgery - Laser trabeculoplasty	-0.44	0.77	.57	-0.03	0.14	.82
Laser Surgery - PRP	-7.19	2.95	.02	0.15	0.55	.78
Laser Surgery - YAG Capsulotomy	-2.69	0.81	.001	-0.22	0.15	.15
Oculoplastic and Orbit	-4.39	2.07	.03	0.19	0.38	.62
Oculoplastic and Orbit - Chalazion Excision	-0.30	0.32	.34	0.03	0.06	.58
Oculoplastic and Orbit - Eyelid laceration	-0.09	0.43	.83	-0.05	0.08	.51
Oculoplastic and Orbit - Ptosis/blepharoplasty	-0.21	0.64	.74	-0.03	0.12	.81
Pterygium/conjunctival and other cornea	-0.49	0.41	.23	0.07	0.08	.36
Retinal Vitreous	-2.27	1.03	.03	-0.17	0.19	.37
Strabismus	0.38	0.81	.64	-0.01	0.15	.95
Total	-41.77	12.89	.001	0.18	2.38	.94
Total Other (Total minus cataract)	-2.69	0.81	.001	-0.22	0.15	.15

© 2025 Culican SM et al. *JAMA Ophthalmology*.

eTable 4: Annual longitudinal change in procedural disparities for URiM residents. Baseline differences included higher numbers of Pterygium/conjunctiva/other cornea and Retina Vitreous cases by URiM trainees. Fewer globe trauma and Retina Vitreous surgeries were reported by URiM trainees annually over time (URiM x Time).

Category	URiM (Basline Difference)			URiM x Time (Difference over Time)		
	Coef	SE	*P*-value	Coef	SE	*P*-value
Cataract	-13.7	8.26	.10	0.94	1.34	.48
Glaucoma - Filtering/shunting procedures	0.59	1.13	.60	-0.11	0.18	.57
Globe Trauma	0.96	0.72	.18	-0.26	0.12	.03
Intravitreal Injection	-3.35	17.08	.84	-2.14	2.77	.44
Keratoplasty	-0.91	0.83	.27	0.20	0.13	.13
Keratorefractive Surgery	-3.87	2.66	.15	0.35	0.43	.42
Laser Surgery - Laser iridotomy	-1.33	1.36	.33	0.17	0.22	.44
Laser Surgery - Laser trabeculoplasty	-0.03	1.81	.99	-0.06	0.29	.84
Laser Surgery - PRP	-8.35	6.92	.23	0.25	1.12	.82
Laser Surgery - YAG Capsulotomy	-0.22	1.91	.91	-0.15	0.31	.63
Oculoplastic and Orbit	-3.75	4.86	.44	-0.16	0.79	.84
Oculoplastic and Orbit - Chalazion Excision	0.36	0.75	.63	-0.10	0.12	.43
Oculoplastic and Orbit - Eyelid laceration	0.66	1.01	.51	-0.23	0.16	.17
Oculoplastic and Orbit - Ptosis/blepharoplasty	-0.20	1.50	.89	-0.17	0.24	.48
Pterygium/conjunctival and other cornea	2.04	0.97	.04	-0.20	0.16	.20
Retinal Vitreous	9.40	2.42	< .001	-1.12	0.39	.004
Strabismus	-3.23	1.90	.09	0.11	0.31	.72
Total	-26.71	30.29	.38	-2.00	4.91	.68
Total Other (Total minus cataract)	-0.22	1.91	.91	-0.15	0.31	.63

© 2025 Culican SM et al. *JAMA Ophthalmology*.

eTable 5: Distribution of active American Board of Ophthalmology Diplomates for those who self-reported gender with certification and practice emphasis. Data from the American Board of Ophthalmology, 2023.

Practice Emphasis	Total	Men	Women	% Women
Cataract	3523	2556	967	27.4%
Comprehensive Ophthalmology	4368	2786	1582	36.2%
Cornea/External Disease	407	258	149	36.6%
Glaucoma	1227	721	506	41.2%
Neuro-Ophthalmology	170	91	79	46.5%
Ocular Oncology	31	21	10	32.3%
Oculoplastic and Orbital Surgery	899	555	344	38.3%
Pediatric Ophthalmology & Strabismus	863	382	481	55.7%
Refractive Surgery	145	124	21	14.5%
Retina – medical & surgery	2630	2114	516	19.6%
Retina – medical only	319	169	150	47.0%
Uveitis/Immunology	70	33	37	52.9%
Other	520	336	184	35.4%
Not Supplied	6950	5921	1029	14.8%
Grand Total	22122	16046	6045	**27.3%**

This supplemental material has been provided by the authors to give readers additional information about their work.

© 2025 Culican SM et al. *JAMA Ophthalmology*.
