# Supplementary material for: Gender Differences in Case Volume Among Ophthalmology Resident Graduates, 2014-2023
Source: JAMA Ophthalmol. 2025 May 1;143(6):490–7. doi: 10.1001/jamaophthalmol.2025.0935 (PMC12046517; doi:10.1001/jamaophthalmol.2025.0935)
Supplement: Supplement 2. — Data Sharing Statement [file jamaophthalmol-e250935-s002.pdf]

Data Sharing Statement

Culican. Gender Differences in Case Volume Among Ophthalmology Resident Graduates 2014-2023. *JAMA Ophthalmol*. Published May 01, 2025. doi:10.1001/jamaophthalmol.2025.0935

Data
Data available: No

Additional Information
Explanation for why data not available: Data must be requested and approved for use by the Accrediation Council for Graduate Medical Education.
